# Supplementary material for: Effect of Enyzmes on the Quality and Predicting Glycaemic Response of Chinese Steamed Bread
Source: Foods. 2023 Jan 6;12(2):273. doi: 10.3390/foods12020273 (PMC9857493; doi:10.3390/foods12020273)
Supplement: Supplementary file 1 [file foods-12-00273-s001.zip › foods-2121599-supplementary.pdf]

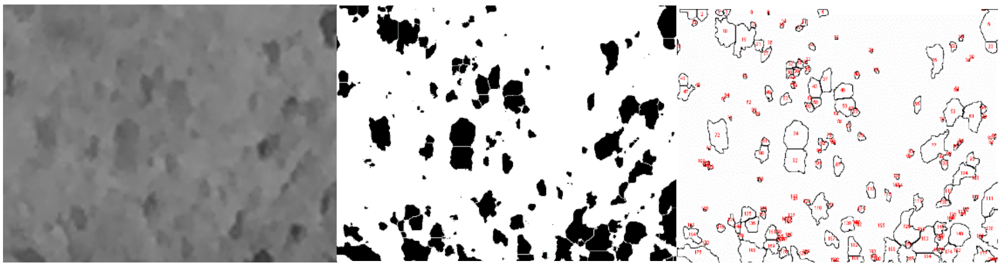

1. **15 % oat bran steamed bread**

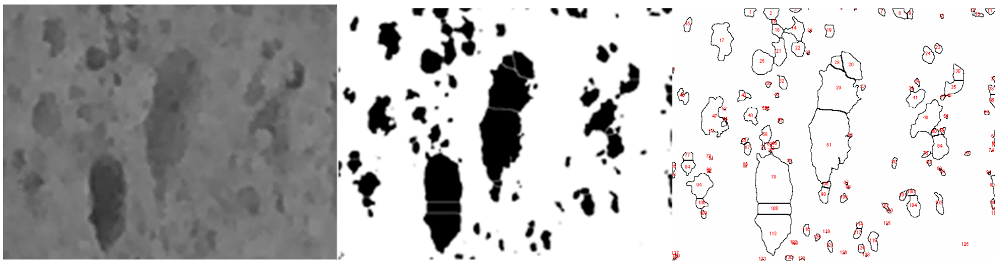

2. **15 % oat bran + 6,70,35ppm steamed bread**

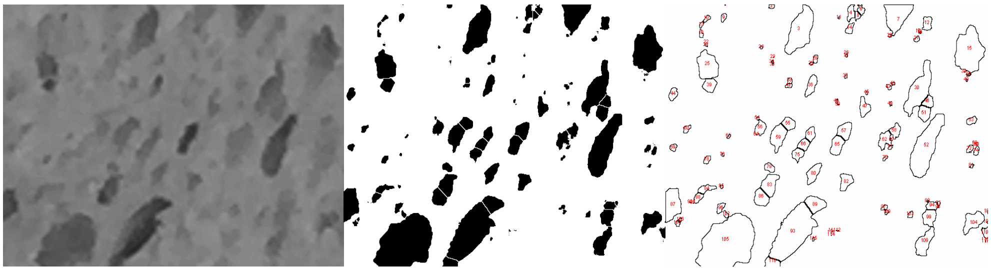

3. **15 % oat bran + 10,120,60ppm steamed bread**

Figure S1. Image analysis of steamed bread slices

Table S1. Description of experimental factors at two level

Factor	(A) α – Amylase	(B) Xylanase	(C) Cellulase
Regular	-	-	-
Control	0	0	0
F1	-1	-1	-1
F2	1	-1	-1
F3	-1	1	-1
F4	1	1	-1
F5	-1	-1	1
F6	1	-1	1
F7	-1	1	1
F8	1	1	1

Amylase (-1, 1) – (6 ppm, 10 ppm); Xylanase (-1, 1) – (70 ppm, 120 ppm); Cellulase (-1, 1) – (35 ppm, 60 ppm).
